# Meta-analysis of the diagnostic and clinical utility of genome and exome sequencing and chromosomal microarray in children with suspected genetic diseases

**DOI:** 10.1038/s41525-018-0053-8

**Published:** 2018-07-09

**Authors:** Michelle M. Clark, Zornitza Stark, Lauge Farnaes, Tiong Y. Tan, Susan M. White, David Dimmock, Stephen F. Kingsmore

**Affiliations:** 1 Rady Children’s Institute for Genomic Medicine, San Diego, CA USA; 2 Murdoch Children’s Research Institute, Melbourne, Australia; 3 Department of Pediatrics, University of California San Diego, San Diego, CA USA; 4 Department of Paediatrics, University of Melbourne, Melbourne, Australia

## Abstract

Genetic diseases are leading causes of childhood mortality. Whole-genome sequencing (WGS) and whole-exome sequencing (WES) are relatively new methods for diagnosing genetic diseases, whereas chromosomal microarray (CMA) is well established. Here we compared the diagnostic utility (rate of causative, pathogenic, or likely pathogenic genotypes in known disease genes) and clinical utility (proportion in whom medical or surgical management was changed by diagnosis) of WGS, WES, and CMA in children with suspected genetic diseases by systematic review of the literature (January 2011–August 2017) and meta-analysis, following MOOSE/PRISMA guidelines. In 37 studies, comprising 20,068 children, diagnostic utility of WGS (0.41, 95% CI 0.34–0.48, *I*^2^ = 44%) and WES (0.36, 95% CI 0.33–0.40, *I*^2^ = 83%) were qualitatively greater than CMA (0.10, 95% CI 0.08–0.12, *I*^2^ = 81%). Among studies published in 2017, the diagnostic utility of WGS was significantly greater than CMA (*P* < 0.0001, *I*^2^ = 13% and *I*^2^ = 40%, respectively). Among studies featuring within-cohort comparisons, the diagnostic utility of WES was significantly greater than CMA (*P* < 0.001, *I*^2^ = 36%). The diagnostic utility of WGS and WES were not significantly different. In studies featuring within-cohort comparisons of WGS/WES, the likelihood of diagnosis was significantly greater for trios than singletons (odds ratio 2.04, 95% CI 1.62–2.56, *I*^2^ = 12%; *P* < 0.0001). Diagnostic utility of WGS/WES with hospital-based interpretation (0.42, 95% CI 0.38–0.45, *I*^2^ = 48%) was qualitatively higher than that of reference laboratories (0.29, 95% CI 0.27–0.31, *I*^2^ = 49%); this difference was significant among studies published in 2017 (*P* < .0001, *I*^2^ = 22% and *I*^2^ = 26%, respectively). The clinical utility of WGS (0.27, 95% CI 0.17–0.40, *I*^2^ = 54%) and WES (0.17, 95% CI 0.12–0.24, *I*^2^ = 76%) were higher than CMA (0.06, 95% CI 0.05–0.07, *I*^2^ = 42%); this difference was significant for WGS vs CMA (*P* < 0.0001). In conclusion, in children with suspected genetic diseases, the diagnostic and clinical utility of WGS/WES were greater than CMA. Subgroups with higher WGS/WES diagnostic utility were trios and those receiving hospital-based interpretation. WGS/WES should be considered a first-line genomic test for children with suspected genetic diseases.

## Genetic sequencing: Improving disease diagnosis in children

Children with suspected genetic disease are more likely to receive a diagnosis when gene sequencing technologies are employed. At present, a microarray test for short pieces of missing or extra chromosomes (chromosomal microarray, CMA) is the recommended test for diagnosing genetic disease in children. The recent use of whole-genome sequencing (WGS) and whole-exome sequencing (WES) for diagnosing genetic disease prompted *Stephen Kingsmore at Rady Children’s Institute for Genomic Medicine in San Diego, California, USA, and colleagues* to examine the effectiveness of these methods compared with CMA. Analyses of studies comprising over 20,000 children showed that using WGS and WES increased the likelihood of diagnosis and led to a change in inpatient management to prevent or ameliorate adverse health outcomes. The authors advocate the use of WGS or WES as a first-line genomic test for genetic disease in children.

## Introduction

Genetic diseases (single-gene disorders, genomic structural defects, and copy number variants) are a leading cause of death in children less than ten years of age.^1–8^ Establishing an etiologic diagnosis in children with suspected genetic diseases is important for timely implementation of precision medicine and optimal outcomes, particularly to guide weighty clinical decisions such as surgeries, extracorporeal membrane oxygenation, therapeutic selection, and palliative care.^9^ With the exception of a few genetic diseases with pathognomonic findings at birth, such as chromosomal aneuploidies, etiologic diagnosis requires identification of the causative molecular basis. In practice, this is remarkably difficult for several reasons: firstly, genetic heterogeneity—there are over 5200 genetic disorders for which the molecular basis has been established.^10^ Secondly, clinical heterogeneity—genetic disease presentations in infants are frequently *formes frustes* of classic descriptions in older children (see, for example Inoue et al.^11^). Thirdly, comorbidity is frequent in infants with genetic diseases—including prematurity, birth trauma, and sepsis—obfuscating clinical presentations.^2^ Fourthly, approximately four percent of children have more than one genetic diagnosis.^12^ Finally, disease progression is faster in children, switching the diagnostic odyssey to a race against time.^9,13,14^

Traditionally, establishment of molecular diagnoses was by serial testing guided by the differential diagnosis. CMA is the recommended first-line genomic test for children with several types of genetic diseases.^15,16^ Serial testing employs many other tests—including newborn screening panels, metabolic testing, cytogenetics, chromosomal fluorescence in situ hybridization, single-gene sequencing, and sequencing of panels of genes associated with specific disease types (such as sensorineural deafness, cardiac dysrhythmias, or epilepsy).^15^ Iterative inquiry of differential diagnoses, however, frequently incurs a diagnostic odyssey and rarely allows etiologic diagnosis in time to influence acute management. Thus, inpatient management of children with suspected genetic diseases largely remains empiric, based on clinical diagnoses.^9^

Over the past five years, WGS and WES have started to gain broad use for etiologic diagnosis of infants and children with suspected genetic diseases.^17–48^ By allowing concomitant examination of all or most genes in the differential diagnosis, WGS and WES have the potential to permit comprehensive and timely ascertainment of genetic diseases. Timely molecular diagnosis, in turn, has the potential to institute a new era of precision medicine for genetic diseases in children. During this period, WGS and WES methods have improved substantially. While numerous studies have been published,^17–48^ there are not yet guidelines for their use by clinicians. Here we report a literature review and meta-analysis of the diagnostic and clinical utility of WGS and WES, compared with CMA, in children (age 0–18 years) with any suspected genetic disease.

## Results

**Fig. 1:**
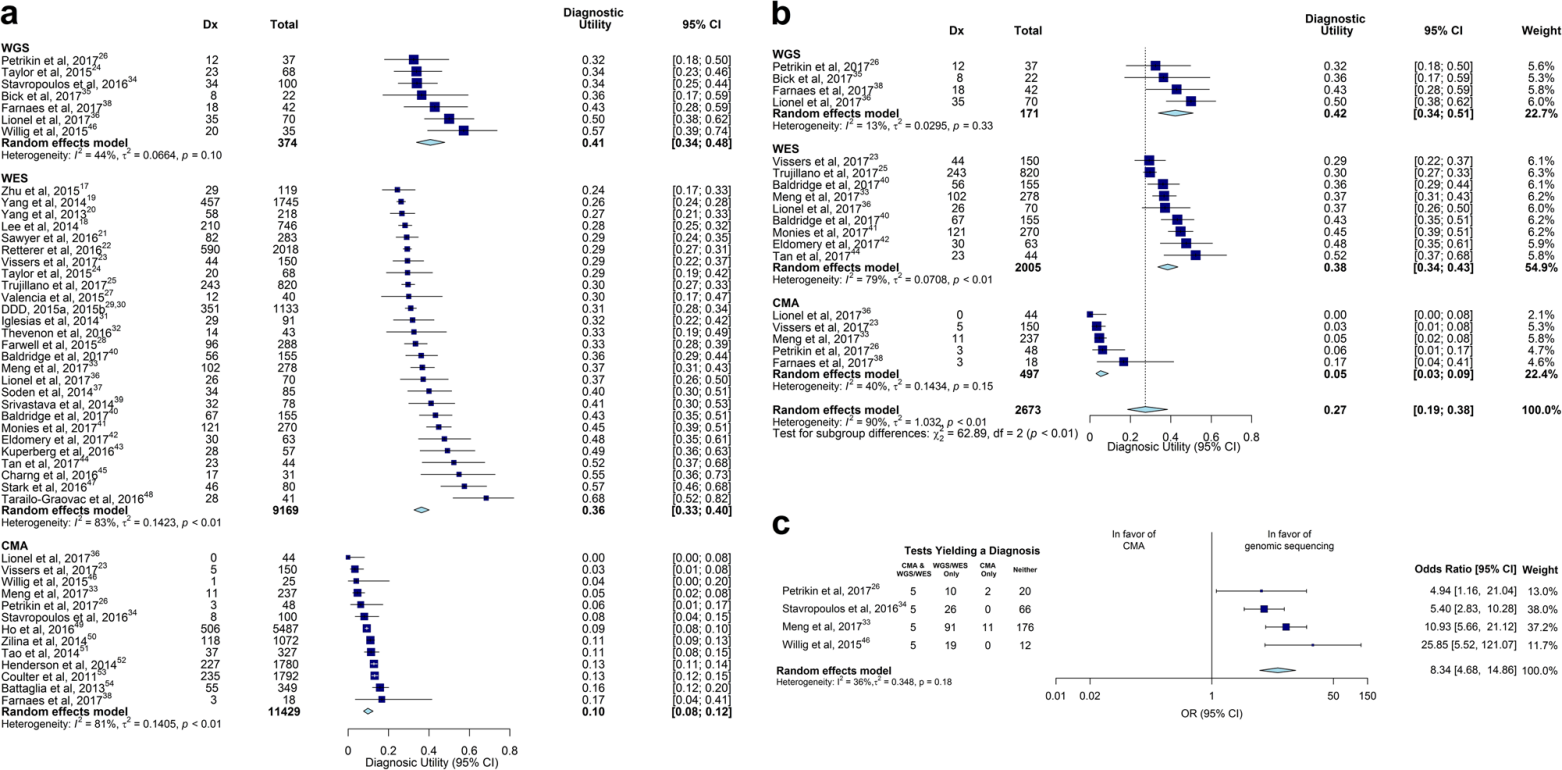
Comparison of diagnostic (Dx) utility of WGS, WES and CMA. **a** The pooled diagnostic utility of WGS and WES were both greater than of CMA. However, severe heterogeneity precluded quantitative analysis. **b** The subset of studies published in 2017 showed reduced heterogeneity for all subgroups. The pooled diagnostic utility with WGS was significantly higher than with CMA (*P* < 0.0001). **c** Among manuscripts that provided complete data for the frequency of diagnoses made by WES and CMA, the pooled odds of diagnosis was 8.3 times greater for WGS (*P* < 0.0001)

WGS and WES are relatively new methods for diagnosis of childhood genetic diseases. We compared the diagnostic utility of WGS and WES with that of CMA, the recommended first-line genomic test for genetic diseases in children with intellectual disability, developmental delay, autism spectrum disorder, and multiple congenital anomalies.^15,16^ A total of 2093 records were identified by searches for studies of the diagnostic utility of WGS, WES, and CMA in affected children with a broad range of suspected genetic diseases (Figure S1). Thirty seven of these, featuring 20,068 children, met eligibility criteria and were included in qualitative analyses (Tables 1 and 2).^17–54^ Thirty-six were case studies; one was a randomized controlled trial.^26^ In these, the pooled diagnostic utility of WGS was 0.41 (95% CI 0.34–0.48, seven studies, 374 children, *I*^2^ = 44%), which was qualitatively greater than WES (0.36, 95% CI 0.33–0.40, 26 studies, *n* = 9014, *I*^2^ = 83%) or CMA (0.10, 95% CI 0.08–0.12, 13 studies, *n* = 11,429, *I*^2^ = 81%, Fig. 1a). Severe heterogeneity (*I*^2^ > 75%) within the WES and CMA groups precluded statistical comparisons.

**Table 1:** Characteristics of studies reporting diagnostic or clinical utility of WES or WGS

Citation	Site	Number of proband children	Genetic diseases tested	Proband age (mean or median)	Reference Lab Or Hospital Or Research Test	WES or WGS	Singleton or Trio	Consanguinity^a^	Molecular diagnosis rate	De novo variant diagnosis rate	Rate of clinical utility^b^
Zhu et al.^17^	US	119	Any	9.5 yr	Research	WES	T	8%	24%	45%	n.d.
Lee et al.^18^	US	520	Any	<18 yr	RefLab	WES	Both^c^	6%	26%	50%	n.d.
Yang et al.^19^	US	1745	Any	6 yr	RefLab	WES	S	n.d.	26%	47%	n.d.
Yang et al.^20^	US	218	Any	<18 yr	Reflab	WES	S	n.d.	27%	47%	n.d.
Sawyer et al.^21^	CA	362	Any	all	Research	WES	Both^c^	21%	29%	n.d.	6%
Retterer et al.^22^	US	3040	Any	7 yr	RefLab	WES	Both^c^	n.d.	29%	43%	n.d.
Vissers et al.^23^	NL	150	Neuro	5 yr 7 mo	RefLab	WES	T	5%	29%	70%	n.d.
Taylor et al.^d^^24^	UK	68	Any	n.d.	Research	WES	Both	17%	29%	n.d.	n.d.
Trujillano et al.^25^	Mixed	820	Any	<15 yr	RefLab	WES	T	45%	30%	24%	n.d.
Petrikin et al.^26^	US	37	Any	<4 mo	H	WGS	T	3%	30%	61%	41%
Valencia et al.^27^	US	40	Any	7 yr	H	WES	T	n.d.	30%	33%	18%
Farwell et al.^28^	US	417	Any	<18 yr	RefLab	WES	Both^c^	n.d.	31%	49%	n.d.
DDD^29,30^	UK	1133	NDD	5.5 yr	Research	WES	T	3%	31%	64%	n.d.
Iglesias et al.^31^	US	91	Any	<18 yr	H	WES	S	11%	32%	41%	18%
Thevenon et al.^32^	FR	43	Neuro	<18 yr	H	WES	S	n.d.	33%	n.d.	9%
Meng et al.^33^	US	178	Any	28 days	H	WES	S	n.d.	33%	n.d.	n.d.
Taylor et al.^d^^24^	UK	68	Any	n.d.	Research	WGS	Both	17%	34%	18%	n.d.
Stavropoulos et al.^34^	CA	100	Any	5.5 yr	H	WGS	S	8%	34%	57%	n.d.
Bick et al.^35^	US	22	Any	<18 yr	H	WGS	S	n.d.	36%	n.d.	27%
Lionel et al.^36^	CA	70	Any	<18 yr	H	WES	S	9%	37%	n.d.	n.d.
Soden et al.^e^^37^	US	85	NDD	7 yr	H	WES	T	5%	40%	50%	21%
Farnaes et al.^38^	US	42	Any	<1 yr	H	WGS	T	2%	40%	38%	31%
Srivastava et al.^39^	US	78	NDD	9 yr	H	WES	S	4%	41%	56%	27%
Baldridge et al.^40^	US	155	Any	6 yr	RefLab + H	WES	S	4%	43%	38%	5%
Meng et al.^33^	US	100	Any	28 days	H	WES	T	n.d.	44%	n.d.	19%
Monies et al.^41^	SA	270	Any	<18 yr	H	WES	S	49%	45%	22%	n.d.
Eldomery et al.^f^^42^	US	63	Any	<18 yr	Research	WES	T	n.d.	48%	n.d.	n.d.
Kuperberg et al.^43^	IS	57	Neuro	<18 yr	H	WES	S	n.d.	49%	61%	9%
Lionel et al.^36^	CA	70	Any	<18 yr	H	WGS	T	9%	50%	n.d.	n.d.
Tan et al.^44^	AU	44	Any	2–18 yr	H	WES	S	n.d.	52%	61%	14%
Charng et al.^45^	SA	31	NDD	all	Research	WES	Both	90%	55%	24%	n.d.
Willig et al.^46^	US	35	Any	26 days	H	WGS	T	3%	57%	65%	37%
Stark et al.^47^	AU	80	Any	8 mo	H	WES	S	21%	58%	35%	23%
Tarailo-Graovac et al.^48^	CA	41	Any	6 yr	Research	WES	T	15%	68%	39%	44%
Sum/Average	**28**	**10392**						**17%**	**31%**	**44%**	**18%**
Range		**22-3040**						**2–90%**	**24–68%**	**18–70%**	**5–44%**

*AU* Australia, *CA* Canada, *IS* Israel, *NDD* neurodevelopmental disabilities, *Neuro* neurologic, *NL* Holland, *SA* Saudi Arabia, *UK* United Kingdom

The statistics in bold are calculated across all rows.

^a^By history or based on long runs of homozygosity

^b^Other than reproductive plans

^c^Statistical difference between S and T within study

^d^Bespoke methods for de novo variants

^e^Corrected to omit infants reported in ref. ^25^

^f^Unsolved by singleton WES

**Table 2:** Characteristics of studies reporting diagnostic or clinical utility of chromosomal microarray

Citation	Site	Number of proband children	Genetic diseases tested	Proband age (mean or median)	Consanguinity	Diagnostic utility	De novo variant diagnosis rate	Rate of clinical utility
Lionel et al.^36^	CA	44	Any	<18 yr	9%	0%	n.d.	0%
Vissers et al.^23^	NL	150	Neuro	5 yr 7 mo	3%	3%	100%	n.d.
Meng et al.^33^	US	237	Any	28 days	n.d.	5%	n.d.	3%
Willig et al.^46^	US	25	Any	26 days	3%	4%	n.d.	0%
Petrikin et al.^26^	US	48	Any	<4 mo	5%	6%	n.d.	2%
Farnaes et al.^38^	US	18	Any	<1 yr	6%	17%	n.d.	6%
Stavropoulos et al.^34^	CA	100	Neuro	5.5 yr	n.d.	8%	n.d.	n.d.
Ho et al.^49^	US	5487	^a^	7.2 yr	n.d.	9%	n.d.	n.d.
Zilina et al.^50^	ES	1072	Any	Postnatal	8%	11%	22%	n.d.
Tao et al.^51^	HK	327	Any	<18 yr	n.d.	11%	n.d.	9%
Henderson et al.^52^	US	1780	^a^	<18 yr	n.d.	13%	n.d.	6%
Coulter et al.^53^	US	1792	^a^	<18 yr	n.d.	13%	n.d.	6%
Battaglia et al.^54^	IT	349	^a^	<18 yr	n.d.	16%	45%	n.d.
Sum/Average	**13**	**11,429**			**7%**	**11%**	**31%**	**6%**

ES Estonia, IT Italy, HK Hong Kong, CA Canada, NL Holland

The statistics in bold are calculated across all rows

^a^Intellectual disability, developmental disorders, autism spectrum disorder, multiple congenital anomalies

### Analysis of heterogeneity of diagnostic utility in studies of WGS, WES, and CMA

**Fig. 2:**
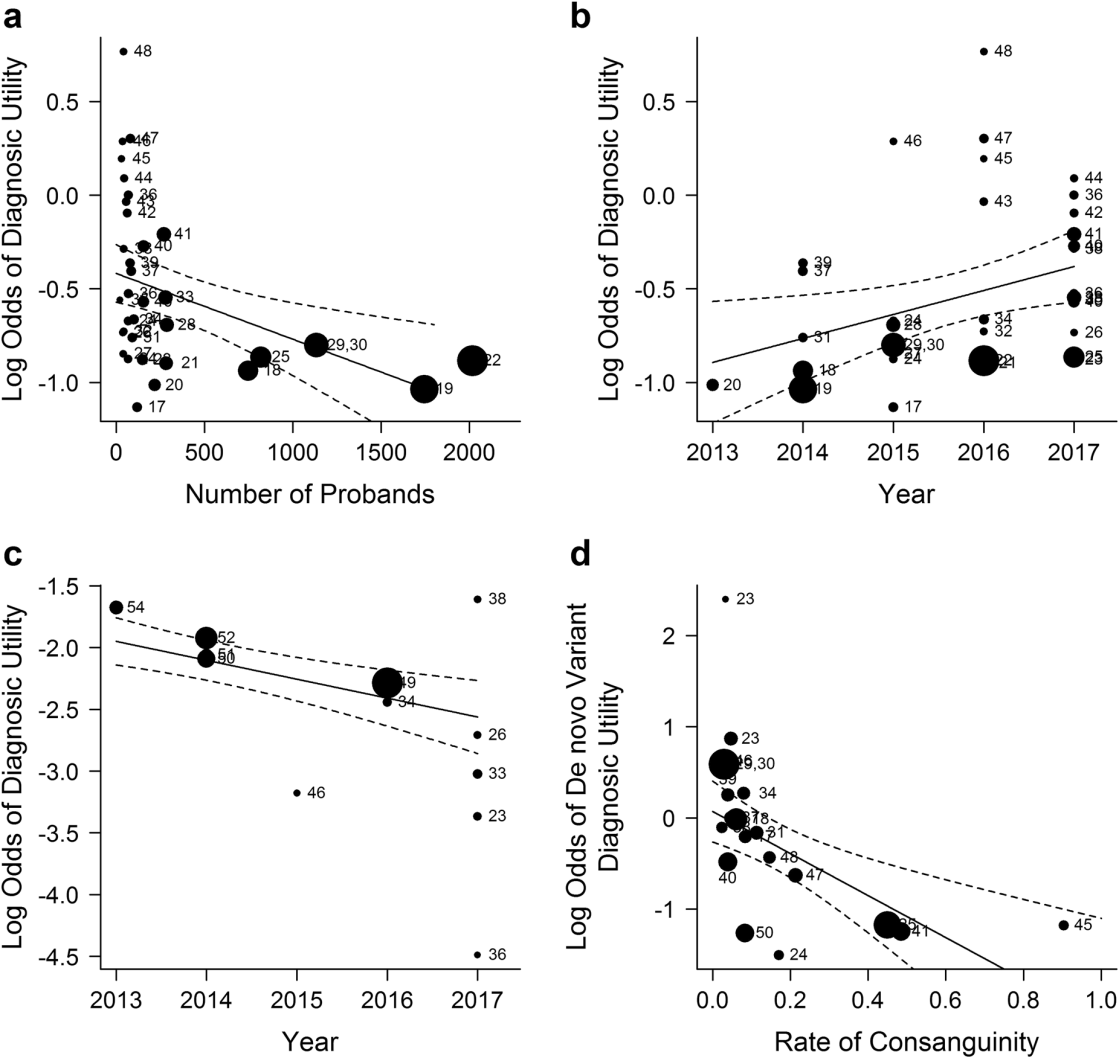
Exploration of heterogeneity of diagnostic utility in WGS and WES studies. **a** Meta-regression scatterplot for study size. On average, an increase of 1000 subjects decreased the odds of diagnosis by 28% (*P* = 0.01). Size of data point corresponds to the study’s inverse-variance weight. **b** Meta-regression scatterplot for diagnostic utility of WGS/WES vs year of study publication. On average, the odds of diagnosis increased by 16% per annum since 2013 (*P* = 0.01). **c** Meta-regression scatterplot for the diagnostic utility of CMA vs year of study publication. The odds of diagnosis decreased by an average of 14% per year between 2013 and 2017 (*P* < 0.001). **d** The rate of diagnosis associated with de novo variation varied inversely with consanguinity. On average, increasing the rate of consanguinity by 10% decreased the odds of de novo variant diagnoses by 21% (*P* < 0.001)

We used meta-regression to model heterogeneity in the diagnostic utility of WGS, WES, and CMA. Studies of WES and WGS varied in size from 22 to 1745 probands; Meta-regression showed a modest relationship between study size and diagnostic utility: on average, an increase of 1000 subjects decreased the odds of diagnosis by 28% (Fig. 2a, *P* = 0.01). Studies were published between 2013 and 2017; meta-regression showed that the odds of diagnosis by WES or WGS increased by 16% each year (Fig. 2c, *P* = 0.01) while the odds of diagnosis by CMA decreased by 14% (Fig. 2c, *P* < 0.001). The rate of consanguinity varied between 0% and 100%. It was not significantly associated with the odds of diagnosis (*P* > 0.05). The proportion of diagnoses in which causal variants occurred de novo (rather than inherited) ranged from 0.18–0.70; meta-regression showed that a 10% increase in the rate of consanguinity in studies of WES and WGS decreased the odds of de novo variant diagnoses by 21% (*P* < 0.001; Fig. 2d). Heterogeneity of diagnostic utility in disease type and proband age subgroups precluded quantitative analysis (Figure S2).

### Subgroup comparisons of diagnostic utility of WGS, WES, and CMA

Heterogeneity within WGS and CMA groups was mild following removal of variance associated with year of publication. In eleven studies of 1962 children published in 2017, the pooled diagnostic utility of WGS (0.42, 95% CI 0.34–0.51, *I*^2^ = 13%) was significantly greater than CMA (0.05, 95% CI 0.03–0.09, *I*^2^ = 40%; *P* < 0.0001, Fig. 1b).^23,25,26,33,35,36,38,40–42,44^

Only two studies, featuring 138 children, compared WES and WGS within cohorts. The diagnostic utility of WES (0.29 and 0.37) did not differ significantly from that of WGS (0.34 and 0.50, respectively; *P* > 0.05).^24,36^ Since the diagnostic utility of WES and WGS was not significantly different, we pooled WGS and WES studies in remaining subgroup analyses. Seven studies directly compared the proportion diagnosed by WGS or WES and CMA in 697 children; in each study, the diagnostic utility of WGS/WES was at least three-fold higher than CMA.^23,26,33,34,36,38,46^ Four of these manuscripts contained enough information to estimate the marginal odds ratios of receiving a diagnosis among subjects that received both WGS/WES and CMA.^26,33,34,46^ In them, the odds of a diagnosis by WGS/WES was 8.3 times greater than CMA (95% CI, 4.7–14.9, *I*^2^ = 36%; *P* < 0.0001, Fig. 1c).

### Comparison of singleton and trio genomic sequencing and effect of site of testing

**Fig. 3:**
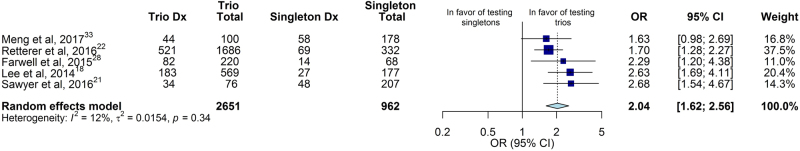
Comparison of diagnostic (Dx) utility of singleton and trio WGS/WES in studies where both analyses were performed. In five studies that conducted within-cohort comparisons of singleton and trio genomic sequencing, the pooled odds of diagnosis for trios was twice that of singletons (*P* < 0.0001)

WGS/WES tests were either of affected probands or trios (proband, mother, father). In eighteen studies, comprising 3935 probands, the heterogeneity of diagnostic utility of singleton and trio WGS/WES was too great to permit quantitative analysis (Figure S3). Meta-analysis was performed in five studies (3613 children) that compared the diagnostic utility of WGS/WES by singleton and trio testing within cohorts.^18,21,22,28,33^ In these studies, the odds of diagnosis using trios was double that using singletons (95% CI 1.62–2.56; *I*^2^ = 12%, *P* < 0.0001, Fig. 3).

**Fig. 4:**
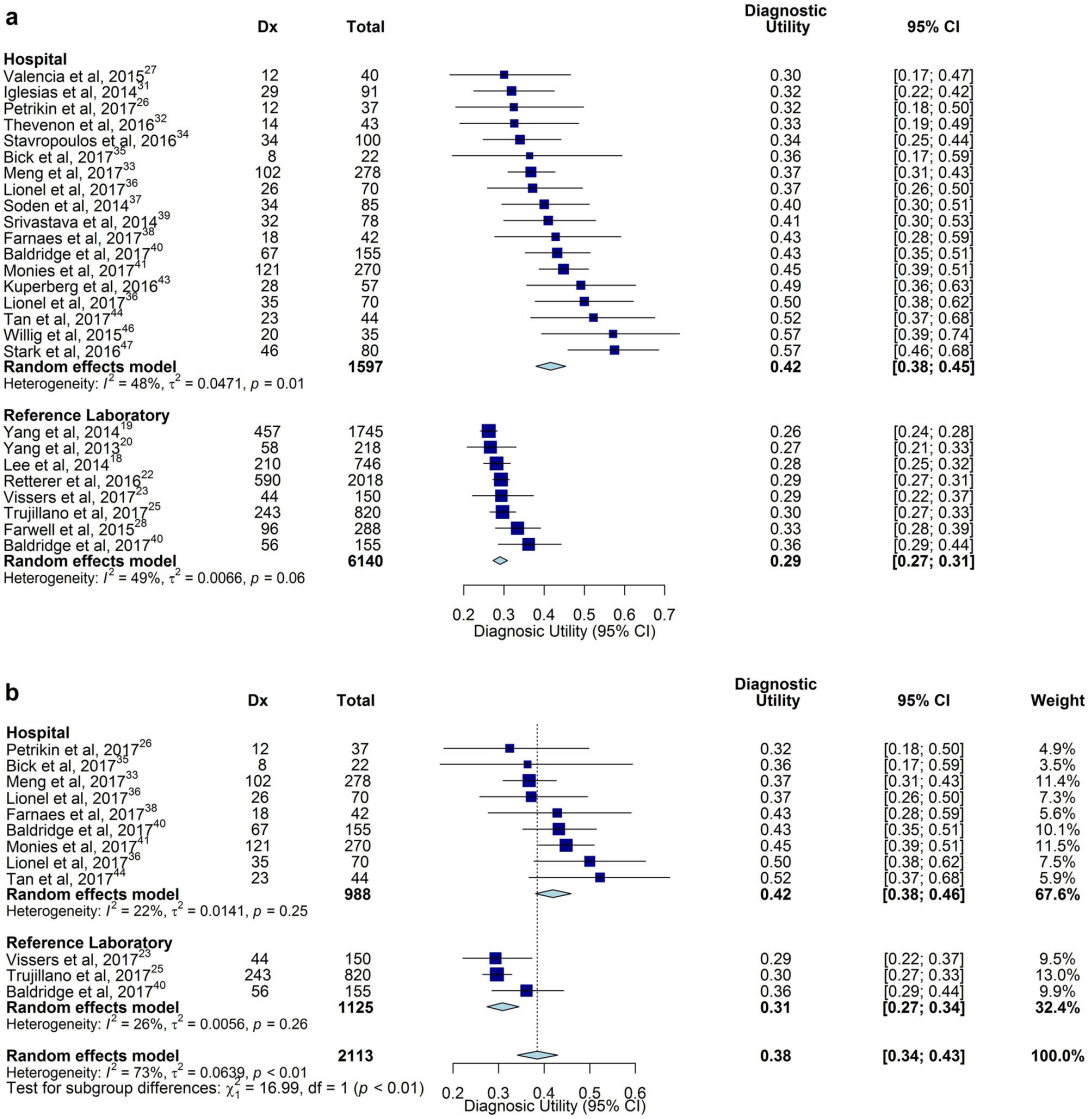
Comparison of diagnostic (Dx) utility of WGS/WES in hospital laboratories and reference laboratories. **a** The pooled diagnostic utility of hospital-based testing was greater than reference laboratory testing. However, substantial heterogeneity was observed. **b** The subset of studies published in 2017 showed reduced heterogeneity for both subgroups. The pooled diagnostic utility was significantly greater in hospitals than in reference laboratories (*P* = 0.004)

Studies were performed in three settings: (i) Research studies of novel methods or disease gene discovery; (ii) Clinical testing with hospital-based interpretation, where a deep phenotype was ascertained from the medical record at interpretation, and clinicopathologic correlation was facilitated by communication between clinicians and interpreters; and (iii) Clinical testing and interpretation in reference laboratories, where phenotype information was limited to that provided in test orders, and communication between clinicians and interpreters was not possible. In nineteen studies, comprising 1597 probands, the diagnostic utility of hospital-based genomic sequencing was 0.42 (95% CI 0.38–0.45, *I*^2^ = 48%), and by reference laboratory-based genomic sequencing was 0.29 (95% CI 0.27–0.31, *I*^2^ = 49%, eleven studies, 6140 probands, Fig. 4a). Both hospital and reference laboratory subgroups demonstrated significant heterogeneity. However, heterogeneity was reduced in ten studies published in 2017 (*I*^2^ = 22%, *P* = 0.25, and *I*^2^ = 26%, *P* = 0.26, respectively).^23,25,26,33,35,36,38,40,41,44^ In these, the diagnostic utility of hospital genomic sequencing was 0.42 (95% CI 0.38–0.46, *I*^2^ = 22%), which was significantly higher than reference laboratories (0.31, 95% CI 0.27–0.34, *I*^2^ = 26%; *P* < 0.0001, Fig. 4b). Of note, hospital studies had an average of 84 subjects, while reference laboratory studies had an average of 558 subjects, providing a possible explanation for the inverse relationship between-study size and rate of diagnosis (Fig. 1a).

### Clinical Utility of WGS, WES, and CMA

**Fig. 5:**
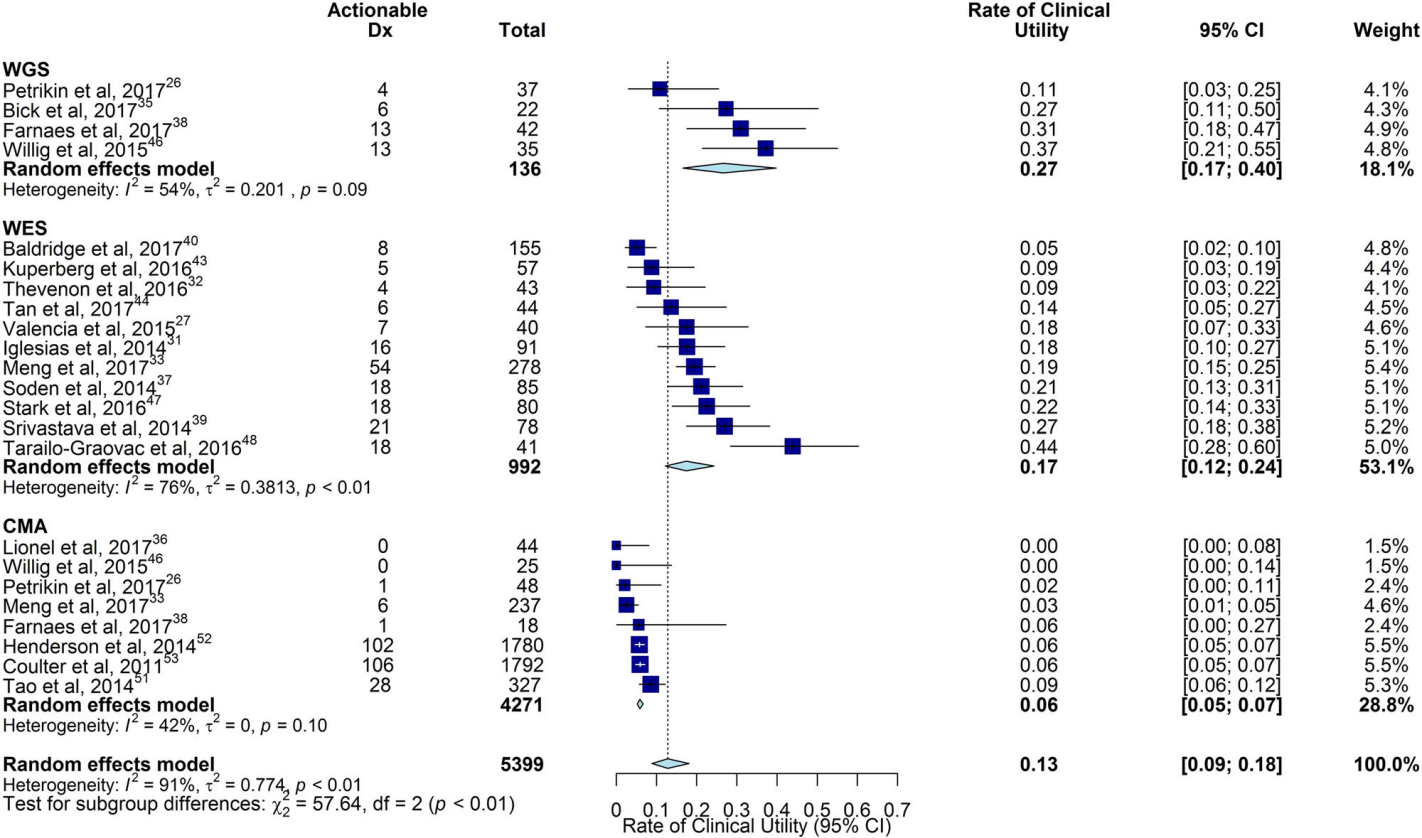
Comparison of the rate of clinical utility of WGS, WES, and CMA. The rate of clinical utility was the proportion of children tested who received a change in medical or surgical management as a result of genetic disease diagnosis. The pooled rate of clinical utility of WGS and WES were both greater than of CMA. However, there was severe heterogeneity in the WES subgroup. Testing for subgroup differences amongst groups with low to moderate heterogeneity, we found that WGS diagnoses lead to an improved rate of clinical utility over CMA diagnoses

To decrease the heterogeneity in definitions of clinical utility between studies, we excluded cases in which the only change in clinical management was genetic counseling or reproductive planning.^55^ The proportion of children receiving a change in clinical management by WGS results was 0.27 (95% CI 0.17–0.40, *I*^2^ = 54%, four studies of 136 children), compared with 0.17 (95% CI 0.12–0.24, *I*^2^ = 76%, twelve studies of 992 children) by WES, and 0.06 (95% CI 0.05–0.07, *I*^2^ = 42%, eight studies of 4271 children) by CMA (Fig. 5). Meta-analysis of WGS and CMA groups, for which heterogeneity was not significant (*P* = 0.09 and *P* = 0.10, respectively), demonstrated that the rate of clinical utility of WGS was higher than CMA (*P* < 0.0001).^26,33,35,36,38,46,51–53^

## Discussion

Current guidelines state that CMA is the first-line genomic test for children with intellectual disability, developmental delay, autism spectrum disorder, and congenital anomalies.^15,49–54,56–58^ Since 2011, WGS and WES have gained relatively broad use for etiologic diagnosis of genetic diseases, but guidelines do not yet exist for their use. A systematic review identified 37 publications in the period January 2011–August 2017, comprising 20,068 affected children, which reported the diagnostic utility of WGS, WES, and/or CMA.^17–54^ Since only thirteen (35%) of these reported results of a comparator test, pooling made comparisons susceptible to confounding from factors including clinical setting, patient factors, eligibility criteria, study quality, clinical expertise, and testing procedures. Meta-regression showed that the odds of diagnosis by WES or WGS increased by 16% each year, while the odds of diagnosis by CMA decreased by 14% each year between 2013 and 2017. For WES and WGS, which are evolving technologies, this likely was due to methodologic improvements; for CMA, which is a mature technology, this was likely due to broader use with time following allowance of reimbursement. Meta-analysis of studies published in 2017, which removed variance associated with year of publication, showed that the diagnostic utility of WGS (0.42, 95% CI 0.34–0.51, *I*^2^ = 13%) was significantly greater than CMA (0.05, 95% CI 0.03–0.09, *I*^2^ = 40%; *P* < 0.0001).^23,25,26,33,35,36,38,40–42,44^ Similarly, meta-analysis of studies featuring within-cohort comparisons showed that the odds of a diagnosis by WGS or WES was 8.3 times greater than CMA (95% CI, 4.7–14.9, *I*^2^ = 36%; *P* < 0.0001).^26,33,34,46^ These results suggest that CMA should no longer be considered the test with highest diagnostic utility for childhood genetic diseases. Rather, WGS or WES should be considered a first-line genomic test for etiologic diagnosis of children with suspected genetic diseases.

While diagnostic utility is an important measure of the value of a clinical test, the relative clinical utility of WGS, WES, and CMA are more relevant for clinicians seeking to improve outcomes of rare childhood genetic diseases through implementation of targeted treatments (precision medicine).^9^ Given the genetic and clinical heterogeneity of genetic disease^10^ and consequent myriad potential therapeutic interventions, it has been difficult to nominate meaningful, generally applicable measures of clinical utility. A previous approach was to collapse all interventions that were temporally and causally related to a molecular diagnosis into an overall “actionability” rate.^26,36,38,46,51–53,55^ Such interventions were either based on practice guidelines endorsed by a professional society or peer-reviewed publications making medical management recommendations. While this has been applied in seven WGS and WES studies to date, definitions of actionability have varied. Furthermore, the evidence base for efficacy of ultra-rare genetic disease treatments is often qualitative rather than quantitative. Nevertheless, after excluding cases in which the only changes were ending the diagnostic odyssey or reproductive planning, WGS and WES had a higher actionability rates than CMA (0.27 [95% CI 0.17–0.40], 0.17 [95% CI 0.12–0.24], and 0.06 [95% CI 0.05–0.07], respectively). This difference was significant for WGS and CMA (*P* < 0.0001), in which within-group heterogeneity was not significant. One caveat was that children tested by CMA in these studies more frequently had multiple congenital anomalies, developmental delay, intellectual disability, or autism spectrum disorders, which were a subset of the presentations of children tested by WGS. Unfortunately, no study has yet reported the relationship between clinical utility of WGS, WES, or CMA and outcomes in children with genetic diseases.

Since WGS is about twice as expensive as WES, which is about twice as expensive as CMA, it is important to identify factors associated with high diagnostic utility. One such factor was the test setting: Hospital laboratory testing had a higher diagnostic utility (0.42, 95% CI 0.38–0.45) than reference laboratory testing (0.29, 95% CI 0.27–0.31). This difference was statistically significant (*P* < 0.0001) among studies published in 2017, in which within-subgroup heterogeneity was not significant. This difference was supported by a study of double interpretation of WES of 115 children, first at a reference laboratory and second at the hospital caring for the children; the diagnostic utility of reference laboratory interpretation was 0.33, and rate of false positive diagnoses was 0.03. The diagnostic utility of hospital interpretation was 0.43, and there were no false positives.^40^ The major difference between hospital and reference laboratory interpretation is the quality and quantity of phenotype information available at time of interpretation. In hospital testing, phenotypic features are ascertained from the medical record, include findings by subspecialist consultants, results of other concomitantly ordered tests, negative findings, and, in difficult cases, are supplemented by discussion with clinicians to ascertain material negative findings or clarify conflicting findings. In reference laboratories, the available phenotypic features are those provided in test orders. They tend to be fewer in number and have less information content. One reference laboratory study found an association between the number of phenotypes available at interpretation and diagnostic yield: the diagnostic utility was 0.26 with one to five phenotype terms, 0.33 with six to fifteen terms, and 0.39 with more than fifteen terms.^25^ This was observed for all phenotypes, family structures, and inheritance patterns. Additional studies are needed to evaluate the reason for the apparent difference in diagnostic utility of hospital and reference laboratory WES/WGS. In the interim, it is suggested that “send out” WES and WGS tests should be accompanied by as much phenotypic information as possible, and open discussion should be encouraged between the laboratory and referring clinician after the results are available to provide a better diagnosis.

De novo variants accounted for the majority of genetic disease diagnoses, except in studies with high rates of consanguinity. Consanguinity is known to increase the population incidence of homozygous recessive genetic diseases. Herein, consanguinity was associated with decreased likelihood of attribution of diagnosis to de novo variants: Meta-regression of 29 studies found the rate of consanguinity to be inversely related to the odds of diagnoses attributed to de novo variants (*P* < 0.001). Consanguinity is thought to increase the diagnostic utility of WGS and WES: In one study, the diagnostic utility of WES was 0.35 in 453 consanguineous families, and 0.27 in 443 non-consanguineous families.^25^ However, meta-analysis failed to show a significant association between the rate of consanguinity and diagnostic utility. Unfortunately, most studies did not report the proportion of probands with a family history of a similar illness, which was also anticipated to increase diagnostic utility.

Testing of parent–child trios is considered superior to singleton (proband) testing for genetic disease diagnosis, since trios facilitate detection of de novo variants and allow phasing of compound heterozygous variants during interpretation (rather than during confirmatory testing). However, trio testing is about twice as costly as singleton testing. Meta-analysis of five studies that compared the diagnostic utility of singleton and trio testing within cohorts showed trio testing to have twice the odds of diagnosis than singleton testing (95% CI 1.62–2.56, *P* < 0.0001).^18,21,22,28,33^ This result was supported by a study in which 36% of unsolved singleton WES cases were diagnosed when re-analyzed as trios.^19,20,42^ Additional studies are needed to guide clinicians with regard to the choice of initial trio or singleton testing. Factors to be considered include cost, time-to-result, and presence of consanguinity or family history of a similar condition.

Clinical WES has been much more broadly used than clinical WGS, since WGS was very expensive until recently, and remains ~$6000 per proband. WES examines almost all known exons and several hundred intronic nucleotides at ends of exons, or approximately two percent of the genome. WGS examines all exons and 90% of the genome. Only seven studies have reported the diagnostic utility of clinical WGS in 374 children.^24,26,34–36,38,46^ Meta-analysis did not show the difference in the diagnostic utility of WGS and WES to be significant. Subsequent to the meta-analysis, one very recent study directly compared the diagnostic utility of clinical WGS and WES in 108 subjects. Three patients (3%) received diagnoses by WGS that were completely unidentified by WES.^59^ Additional studies are needed since the diagnostic utility of WGS and WES are increasing disparately as a result of improved identification of disease-causing copy number and structural variations, repeat expansions, and non-exonic regulatory and splicing variations.^34,36,42,57,58,60–64^ In one recent study, these increased diagnostic utility by 36%.^42^ Recent research has shown WGS to have higher analytic sensitivity for copy number and structural variations than CMA, particularly small structural variations (less than 10,000 nucleotides^34,36,64^), suggesting that WGS may become the single first-line genomic test for etiologic diagnosis of most children suspected to have a genetic disease. However, the published data do not yet support superiority of WGS over WES.

This meta-analysis had several limitations. We used published diagnostic rates at face value; we did not reclassify diagnoses according to the strength of evidence of gene-disease relationships.^65^ Comparisons should be interpreted with caution due to heterogeneity of pooled averages of the published data. We were unable to control for heterogeneity in diagnostic utility associated with different types of clinical presentations or “cherry picking” (enrichment for children considered a priori to have high likelihood of a genetic etiology of disease. The highest level of evidence for clinical interventions is meta-analyses of randomized controlled trials (Level I).^66^ For WGS and WES, only one such study has yet been published.^26^ Published studies constitute Level II evidence (controlled studies or quasi-experimental studies) and Level III evidence (non-experimental descriptive studies, such as comparative studies, correlation studies, and case-control studies). The meta-analysis did not include diagnostic specificity (which has only been directly examined in one manuscript),^40^ nor the relative cost-effectiveness of WGS, WES, and CMA, either in terms of the cost of the diagnostic odyssey or long-term impact on healthcare utilization. It excluded next-generation sequencing-based panel tests, which are frequently used for specific presentations, such as epilepsy. It did not include subgroup analysis of the diagnostic utility or clinical utility by affected organ system, which might have identified subgroups of children who are most likely to benefit from testing. While, on average, the CMA studies were one or two years older than the WGS/WES studies, the diagnostic utility of CMA did not increase with time. In several of the WGS/WES studies, patients had previously received negative CMA tests, diminishing the relative diagnostic utility of WGS/WES.

## Conclusions

In meta-analyses of 37 studies of children with suspected genetic diseases, the diagnostic utility of WGS (0.41, 95% CI 0.34–0.48) and WES (0.36, 95% CI 0.33–0.40) were higher greater than CMA (0.10, 95% CI 0.08–0.12), the current first-line genomic test for certain childhood genetic disorders. The same was true for the rate of clinical utility (WGS 0.27, 95% CI 0.17–0.40; WES 0.17, 95% CI 0.12–0.24; CMA 0.06, 95% CI 0.05–0.07). Additional randomized controlled studies are needed, particularly studies that examine the diagnostic determinants of optimal outcomes for children with rare genetic diseases.^67^

## Methods

### Data sources and record identification

We searched PubMed from 1 January 2011, to 4 August 2017 with the terms (“exome sequencing” or “whole-genome sequencing” or “chromosomal microarray”), and (“diagnosis” or “clinical”), and “genetic disease” (Figure S1). We manually searched journals not indexed by PubMed that published articles related to clinical genomic testing. There were no language restrictions.

### Study screening and eligibility

Studies that evaluated the diagnostic utility (proportion of patients tested who received genetic diagnoses) or clinical utility (proportion of patients tested in whom the diagnosis changed medical or surgical management) of WGS, WES, and/or CMA were eligible. We limited eligibility to studies of cohorts with a broad range of genetic diseases, rather than one or a few disease types or clinical presentations, and in which the majority of probands were less than 18 years old. The systematic review and meta-analysis were performed according to the MOOSE and PRISMA guidelines (Table S1 and Figure S1).

### Inclusion criteria and data extraction

Data extraction was manual. Data were reviewed for completeness and accuracy by at least two expert investigators and disparities were reconciled by consensus. The QUADAS-2 tool was used to assess the quality of the included studies (Table S2). The PICOTS typology of the criteria for inclusion of studies in quantitative analyses was:

**Patients**: Data extraction was limited to affected children (age less than 18 years) with suspected genetic disease.

**Intervention**: WGS, WES, and/or CMA for etiologic diagnosis of a suspected genetic disease.

**Comparator**: The groups compared were subjects tested by WGS, WES, and CMA. CMA was treated as the Reference Standard. Subgroups were patients tested with WGS, WES, or CMA as singletons (proband) and trios (parents and child). Trios did not include the use of parental DNA for confirmatory phasing by Sanger sequencing.

**Outcomes**: Diagnostic utility, rate of clinical utility. Molecular diagnoses were defined as pathogenic or likely pathogenic diplotypes (pairs of haplotypes) affecting genes or genomic variations with definitive, strong, or moderate associations with phenotypes that overlapped at least part of the clinical features of the affected patient, and that were reported to the patient’s clinician.^65^ Variants of uncertain significance and secondary findings were not extracted. The definition of clinical utility conformed to a position statement of the American College of Medical Genetics and Genomics, but was limited to changes in management for individual patients.^55^

**Timing**: Where more than one publication reported results from a cohort, we included the most recent value for diagnostic utility. Clinical utility was assessed acutely (typically within six months of enrollment of the last patient).

**Settings**: Testing was performed clinically in hospital laboratories and reference laboratories, and experimentally in research laboratories. Hospital and reference laboratory clinical tests were defined primarily by the site of testing and, as disclosed in the methods, and, secondarily, by the affiliations of the authors. Clinical testing was defined as testing under fixed protocols that were attested to comply with state or national regulatory guidelines for in vitro diagnostic testing. Experimental research tests were those that explored the utility of novel or bespoke methods of testing or analysis.

**Study Design**: There were no study design restrictions.

### Statistical Analysis

Between-study heterogeneity was explored by univariate analysis. Potential sources of heterogeneity included year of publication, number of probands, genetic disease tested, and consanguinity. The variable for genetic disease tested was treated as having four categories in the publications examined: any genetic disease, genetic diseases that included neurodevelopmental and metabolic disorders, neurodevelopmental disabilities alone, and infants (average proband age less than one year at testing). The effect of disease tested on heterogeneity was explored with a random-effects model as described below. We used meta-regression to study associations of continuous variables (year, study size, and the rate of consanguinity) and heterogeneity.

When comparing rates between studies, raw proportions (i.e., molecular diagnostic and clinical utility rates) for individual studies were logit transformed due to small sample sizes and low event rates.^68^ For each comparison, only the relevant subsets of patients reported in each relevant study were retained. Pooled subgroup proportions and their variances were obtained by fitting an inverse-variance weighted logistic-normal random-effects model to the data. 95% confidence intervals (CIs) for individual studies were derived using the Clopper–Pearson exact method.^69^ Pooled proportions and CIs were back-transformed for interpretation. For studies which conducted within-cohort comparisons, an inverse-weighted random-effects model was used to estimate pooled odds ratios (ORs). Due to the paired nature of the data, the marginal cross-over OR estimator of Becker and Balagtas^70,71^ was used for the meta-analysis of studies that conducted within-cohort comparisons of WES and CMA diagnostic rates. For all analyses, between-study heterogeneity was assessed using between-study variance (τ^2^), the *I*^2^ statistic^72^ and Cochran’s Q test.^73^ *I*^2^ values of 25, 50, and 75% indicate mild, moderate, and severe heterogeneity, respectively.^72^ Subgroup analyses were conducted to minimize severe heterogeneity between studies. Subgroup differences in rates and ORs were tested when there was not significant evidence of within-group heterogeneity. Forest plots were used to summarize individual study and pooled group meta-analysis statistics. Two-tailed *P* ≤ 0.05 were considered statistically significant. All statistical analyses were conducted using the ‘meta’ (version 4.8.1) and ‘metafor’ (version 2.0.0) packages in R (version 3.3.3).^74–76^

## Electronic supplementary material

Supplemental Material
