# Supplementary material for: Meta-analysis of the diagnostic and clinical utility of genome and exome sequencing and chromosomal microarray in children with suspected genetic diseases
Source: NPJ Genom Med. 2018 Jul 9;3:16. doi: 10.1038/s41525-018-0053-8 (PMC6037748; doi:10.1038/s41525-018-0053-8)

**Supplementary Appendix**

**Meta-analysis of genomic sequencing in infants and children with likely genetic diseases demonstrates diagnostic and clinical utility**

Michelle M. Clark PhD^1^, Zornitza Stark DM^2^, Lauge Farnaes MD PhD^1,3^, Tiong Y. Tan MD PhD^2,4^, Susan M. White MD^2,4^, David Dimmock MD^1^, Stephen F. Kingsmore MB ChB BAO DSc^1^

Affiliations:

1. Rady Children’s Institute for Genomic Medicine, San Diego, CA, USA;
2. Murdoch Children’s Research Institute, Melbourne, Australia;
3. Department of Pediatrics, University of California San Diego, San Diego, CA, USA;
4. Department of Paediatrics, University of Melbourne, Melbourne, Australia.

**Supplementary Methods**

The MOOSE (Meta-analyses Of Observational Studies in Epidemiology), PRISMA (Preferred Reporting Items for Systematic Reviews and Meta-Analyses), and PICOTS (populations, interventions, comparators, outcomes, timing and setting) and QUADAS-2 guidelines and tools were from references S1-S4, respectively.

S1. Stroup DF, Berlin JA, Morton SC, Olkin I, Williamson GD, Rennie D, et al. Meta-analysis of observational studies in epidemiology: a proposal for reporting. Meta-analysis Of Observational Studies in Epidemiology (MOOSE) group. JAMA 2000;283:2008–12.

S2. Moher D, Liberati A, Tetzlaff J, Altman DG; PRISMA Group. Preferred reporting items for systematic reviews and meta-analyses: the PRISMA statement. PLoS Med. 2009 Jul 21;6(7):e1000097.

S3. Thompson M, Tiwari A, Fu R, et al. A Framework To Facilitate the Use of Systematic Reviews and Meta-Analyses in the Design of Primary Research Studies [Internet]. Rockville (MD): Agency for Healthcare Research and Quality (US); 2012 Jan. <https://www.ncbi.nlm.nih.gov/books/NBK83626/>

S4. Whiting PF, Rutjes AW, Westwood ME, Mallett S, Deeks JJ, Reitsma JB, Leeflang MM, Sterne JA, Bossuyt PM; QUADAS-2 Group. QUADAS-2: a revised tool for the quality assessment of diagnostic accuracy studies. Ann Intern Med. 2011 Oct 18;155(8):529-36.

**Table S1: MOOSE (Meta-analysis Of Observational Studies in Epidemiology) Checklist**

| **Item No** | **Recommendation** | **Reported on Page** |
| --- | --- | --- |
| Reporting of background should include | | |
| 1 | Problem definition | 4 |
| 2 | Hypothesis statement | 3 |
| 3 | Description of study outcome(s) | 5 |
| 4 | Type of exposure or intervention used | 5 |
| 5 | Type of study designs used | 5 |
| 6 | Study population | 5 |
| Reporting of search strategy should include | | |
| 7 | Qualifications of searchers | 5 |
| 8 | Search strategy, including time period included in the synthesis and key words | 5 |
| 9 | Effort to include all available studies, including contact with authors | 5 |
| 10 | Databases and registries searched | 5 |
| 11 | Search software used, name and version, including special features used | 5 |
| 12 | Use of hand searching (eg, reference lists of obtained articles) | 5 |
| 13 | List of citations located and those excluded, including justification | Figure S1 |
| 14 | Method of addressing articles published in languages other than English | 5 |
| 15 | Method of handling abstracts and unpublished studies | 5 |
| 16 | Description of any contact with authors | 5 |
| Reporting of methods should include | | |
| 17 | Description of relevance or appropriateness of studies assembled for assessing the hypothesis to be tested | 5 |
| 18 | Rationale for the selection and coding of data | 5 |
| 19 | Documentation of how data were classified and coded | 5 |
| 20 | Assessment of confounding | 5 |
| 21 | Assessment of study quality, including blinding of quality assessors, stratification or regression on possible predictors of study results | 5 |
| 22 | Assessment of heterogeneity | 5 |
| 23 | Description of statistical methods in sufficient detail to be replicated | 5 |
| 24 | Provision of appropriate tables and graphics | Multiple |
| Reporting of results should include | | |
| 25 | Graphic summarizing individual study estimates and overall estimate | Multiple |
| 26 | Table giving descriptive information for each study included | Table S2 |
| 27 | Results of sensitivity testing (eg, subgroup analysis) | Multiple |
| 28 | Indication of statistical uncertainty of findings | Multiple |

| Reporting of discussion should include | | |
| --- | --- | --- |
| 29 | Quantitative assessment of bias | 9 |
| 30 | Justification for exclusion | 9 |
| 31 | Assessment of quality of included studies | 10 |
| Reporting of conclusions should include | | |
| 32 | Consideration of alternative explanations for observed results | 9 |
| 33 | Generalization of the conclusions | 10 |
| 34 | Guidelines for future research | 10 |
| 35 | Disclosure of funding source | 11 |

**Table S2: Study Characteristics**

| **Citation** | **Site** | **Study Design** | **Study Outcomes** | **Total Study Size (adults and children)** | **Population** |
| --- | --- | --- | --- | --- | --- |
| Baldridge et al, 2017 ^40^ | US | Case series | Diagnostic yield of WES before and after assessment by a medical geneticist, incorporation of detailed phenotypic and molecular data, and utilization of additional diagnostic modalities. Effect of WES results on auxiliary tests, management, and research studies. | 155 | Patients who underwent clinical exome sequencing in the Washington University School of Medicine Exome Clinic between March 2012 and January 2015 identified by retrospective chart review |
| Battaglia et al, 2013 ^54^ | IT | Case series | Diagnostic yield of CMA | 349 | Recruited patients affected by DD/ID/ASDs/dysmorphic features of unknown origin observed at the Stella Maris Institute between May 2004 and December 2011 |
| Bick et al, 2017 ^35^ | US | Case series | Rates of diagnosis of WGS, incidental findings, and impact of diagnosis on patient management | 22 | Patients with suspected genetic disorders in the Genetics Clinic at Children's Hospital Wisconsin recommended for WGS by a case review team |
| Charng et al, 2016 ^45^ | SA | Case series | WES molecular diagnosis rate | 31 | Probands and their siblings and parents from consanguineous families with brain malformation and/or developmental delay/intellectual disability from Saudi Arabia |
| Coulter et al, 2011 ^53^ | US | Case series | Rate of clinically relevant results and rate of change in medical management | 1792 | Patients at Children's Hospital Boston with DD/ID, ASD, and congenital anomalies who received CMA testing performed from July 1, 2009, to July 1, 2010 identified by retrospective chart review |
| DDD, 2015a, 2015b ^29,30^ | UK | Case series | Diagnostic yield of exome sequencing and array-based detection of chromosomal rearrangements | 1133 | Children with diverse, severe undiagnosed developmental disorders, through all 24 regional genetics services of the UK National Health Service and Republic of Ireland recruited with parents |
| Eldomery et al, 2017 ^42^ | US | Case series | Molecular diagnostic yield of WES using parent-offspring trios and singletons | 74 | Children who received clinical diagnostic WES at Baylor Genetics laboratory and for whom a molecular diagnosis was not achieved at the time of initial reporting |
| Farnaes et al. 2017 ^38^ | US | Case series | Rate of implementation of WGS-associated precision medicine and impact on health outcomes and healthcare utilization | 42 | Children less than one year of age who were inpatient at Rady Children's Hospital San Diego at the time of enrollment and received WGS |
| Farwell et al, 2015 ^28^ | US | Case series | WES yield of overall positive and novel gene findings in families with undiagnosed genetic conditions. Phenotypes of cases with highest diagnostic rates and inheritance patters among positive findings. Diagnostic rate from testing trios compared to singletons. | 500 | Patients ascertained sequentially through clinical samples sent to Ambry Genetics Laboratory for diagnostic exome sequencing beginning in September 2011 |
| Henderson et al, 2014 ^52^ | US | Case series | Rate of diagnosis and impact of chromosomal microarray on clinical management | 1780 | Patients with multiple congenital anomalies, developmental delay, intellectual disability, and autism spectrum disorders who had abnormal chromosomal microarray findings reported by Cytogenetics Laboratory between August 2009 and August 2012 identified by retrospective review of the EMR |
| Ho et al, 2016 ^49^ | US | Case series | Detection of CNVs by chromosomal microarray analysis and corresponding effect on medical management. Association of detection rate and pathogenic yield of CMA with primary indications for testing, age, specialty of ordering doctor, and resolution of array | 5487 | Patients referred for CMA to Lineagen (a CLIA-licensed laboratory) for etiological diagnosis of DD/ID/ASD and MCAs between July 2012 and December 2015 |
| Iglesias et al, 2014 ^31^ | US | Case series | Rate of definitive diagnosis, effect of results on phenotype expansion and identification of new candidate disease genes, and clinical utility of whole-exome sequencing | 115 | Patients who were clinically evaluated by a board-certified clinical geneticist and a board-certified genetic counselor at Columbia University Medical Center from October 2011 to July 2013 and for whom WES had been completed in that time period identified by retrospective chart review |
| Kuperberg et al, 2016 ^43^ | IS | Case series | Overall diagnostic rate, clinical characteristics of diagnosed patients, and clinical utility of WES | 57 | Pediatric neurological patients of MAGEN clinic at Wolfson Medical Center between 2011 and 2015 who were suspected as having a monogenic disorder but remained undiagnosed after extensive testing |
| Lee et al, 2014 ^18^ | US | Case series | Clinical indications for Clinical exome sequencing (CES) requests, molecular diagnostic rates of CES overall and for phenotypic subgroups, and differences in molecular diagnostic rates between trio-CES and proband-CES | 814 | Patients with undiagnosed, suspected genetic conditions who received clinical exome sequencing at the University of California, Los Angeles, Clinical Genomics Center between January 2012 and August 2014 |
| Lionel et al, 2017 ^36^ | CA | Case series | Diagnostic yield of WGS compared with targeted gene sequencing panels | 103 | Patients from pediatric non-genetic subspecialty clinics at The Hospital for Sick Children, Toronto recruited between April 2013 to June 2015, each with a clinical phenotype suggestive of an underlying genetic disorder |
| Meng et al, 2017 ^33^ | US | Case series | Indications for testing, diagnostic yield of clinical exome sequencing, turnaround time, molecular findings, and impact on medical management | 278 | Unrelated infants who received clinical exome sequencing within the first 100 days of life admitted to Texas Children’s Hospital in Houston between December 2011 and January 2017. |
| Monies et al, 2017 ^41^ | SA | Case series | Diagnostic yield of panels and WES, the distribution of disease-mutations, effect on phenotypic expansion, and the impact on management | 1000 | The first 1000 families who were referred to the only clinical next-generation sequencing lab in Saudi Arabia with a wide-range of suspected Mendelian phenotypes |
| Petrikin et al, 2017 ^26^ | US | Randomized Control Trial | Rate of genetic diagnosis within 28 days of enrollment or first standard test order | 65 | Infants aged less than four months, in neonatal and pediatric intensive care units at Children’s Mercy – Kansas City, and with illnesses of unknown etiology enrolled between October 2014 - June 2016 |
| Retterer et al, 2016 ^22^ | US | Case series | Overall diagnostic yield of WES for many different clinical indications, most common clinical phenotypes among diagnosed patients, and the effect of using trios compared to proband-only testing on the diagnostic yield | 3040 | Cases referred to GeneDx for clinical WES from January 2012 until October 2014 |
| Sawyer et al, 2016 ^21^ | CA | Case series | Identification of mutations in genes known to cause disease and novel gene discovery using WGS | 362 | Patients enrolled in the FORGE project ascertained from 21 participating academic centers across the country and who remained undiagnosed after appropriate clinical and molecular investigations |
| Soden et al, 2014 ^37^ | US | Case series | Rate of diagnosis of WES, financial impact of genomic diagnoses, and clinical impact | 85 | Families with one or more undiagnosed children with NDDs suspected of having a monogenetic disease followed in ambulatory clinics at Children's Mercy – Kansas City |
| Srivastava et al, 2014 ^39^ | US | Case series | Overall presumptive diagnostic rate, inheritance pattern, identification of common clinical phenotypes among diagnosed patients, changes in clinical management | 78 | Patients who presented to the pediatric neurogenetics clinic at Kennedy Krieger Institute for etiological evaluation of previously unexplained neurodevelopmental disorders from November 2011 through February 2014 and received clinical WES |
| Stark et al, 2016 ^47^ | AU | Case series | The diagnosis rate, clinical utility, and impact on management of singleton WES | 80 | Infants with multiple congenital abnormalities and dysmorphic features or other features strongly suggestive of monogenic disorders recruited during clinical care by the genetics service at the Royal Children’s Hospital, Melbourne, Australia, between February 2014 and May 2015 |
| Stavropoulos et al, 2016 ^34^ | CA | Case series | Change in diagnostic yield of for mutation detection of WGS compared to standard genetic testing including chromosomal microarray | 100 | 100 consecutive patients at The Hospital for Sick Children, Toronto referred to a pediatric genetics service who met criteria for CMA |
| Tan et al, 2017 ^44^ | AU | Case series | The clinical utility of a molecular diagnosis and the cost-effectiveness of alternative diagnostic trajectories, depending on timing of WES | 44 | Children older than 2 years suspected of having a monogenic disorder prospectively recruited from May 1 through November 30, 2015 after referral from general and subspecialist pediatricians at Royal Children’s Hospital, Melbourne, Australia |
| Tao et al, 2014 ^51^ | HK | Case series | Microarray detection rate of pathogenic/likely pathogenic findings and impact of CMA results on medical management | 327 | Children who received CMA due to unexplained intellectual disability/developmental delay, autism spectrum disorders, and/or multiple congenital anomalies from Queen Mary Hospital and the Duchess of Kent Children’s Hospital between January 2011 and May 2013 |
| Tarailo-Graovac et al, 2016 ^48^ | CA | Case series | Diagnostic yield, effect on clinical management, and identification of disease genes using deep phenotyping and WES | 41 | Patients from BC Children's Hospital with confirmed or potential intellectual developmental disorder along with a metabolic phenotype of unknown cause after comprehensive clinical phenotyping with extensive previous metabolic or genetic testing |
| Taylor et al, 2015 ^24^ | UK | Case series | Number of candidate variants identified using different strategies for variant calling, filtering, annotation and prioritization | 68 | Patients in whom WGS findings could have immediate clinical utility in terms of diagnosis, prognosis, treatment selection, or genetic counselling and reproductive choices recruited as part of a collaboration between the Wellcome Trust Centre for Human Genetics at the University of Oxford, the Oxford NIHR Biomedical Research Centre and Illumina Inc. |
| Thevenon et al, 2016 ^32^ | FR | Case series | Diagnostic yield, clinical utility, and feasibility of WGS | 43 | Children overcoming a ‘diagnostic odyssey’ with severe to profound non-syndromic ID and/or an EE recruited from The Reference Center for Developmental Anomalies and Malformative Syndromes |
| Trujillano et al, 2017 ^25^ | Mixed | Case series | Variants detected with clinical WES, WES diagnostic yield, phenotype complexity, and validating novel genes | 1000 | 1000 consecutive, unrelated patients with suspected Mendelian disorders from 54 countries referred to Centogene for diagnostic WES between January 2014 and January 2016 |
| Valencia et al, 2015 ^27^ | US | Case series | Diagnoses using exome data, altered management of patients, secondary findings, and cost analysis | 40 | Pediatric patients referred for exome sequencing at Cincinnati Children’s Hospital Medical Center who had undergone extensive diagnostic evaluations that did not lead to a unifying diagnosis |
| Vissers et al, 2017 ^23^ | NL | Case series | Clinical utility of whole-exome sequencing (WES) in complex pediatric neurology in terms of diagnostic yield and costs compared to conventional genetic testing | 150 | Patients with nonacute neurological symptoms of suspected genetic origin in the Department of Pediatric Neurology at the Radboud University Medical Center referred by pediatricians, neurologists, and psychiatrists for further diagnostics |
| Willig et al, 2015 ^46^ | US | Case series | Comparison of WGS and standard testing in terms of rate of diagnosis, time to diagnosis, clinical usefulness, and palliative care | 35 | Infants enrolled in a research biorepository who had genomic sequencing and standard diagnostic tests to diagnose monogenic disorders of unknown cause in the affected children at Children's Mercy – Kansas City |
| Yang et al, 2013 ^20^ | US | Case series | Diagnoses based on WES and inheritance pattern of findings in patients with suspected genetic disorders | 250 | Patients with a whole-exome sequencing test ordered by their physician from the Whole Genome Laboratory of Baylor College of Medicine starting in October 2011 |
| Yang et al, 2014 ^19^ | US | Case series | Whole-exome sequencing diagnosis rate overall and by phenotypic category, mode of inheritance, spectrum of genetic events, and reporting of incidental findings. | 2000 | 2000 consecutive, unrelated patient cases in this study who were referred from physicians starting in June 2012 through November 2013 for clinical whole-exome sequencing at the Whole Genome Laboratory of Baylor College of Medicine |
| Zhu et al, 2015 ^17^ | US | Case series | Rate of diagnosis based on known gene-disease associations and identification of bioinformatic signatures to identify novel disease genes | 119 | Patients recruited at the Genome Sequencing Clinic at Duke University Medical Center and the pediatric clinic of the Sheba Medical Center in Tel HaShomer, Israel with a severe clinical presentation and had an undiagnosed genetic disorder or were suspected to have a specific genetic disorder that was genetically unresolved based on known diagnostic panels |
| Zilina et al, 2014 ^50^ | ES | Case series | CMA yield of pathogenic/ likely pathogenic and VUS findings when used for prenatal and postnatal diagnosis | 1191 | Prenatal and postnatal patients at Tartu University Hospital with an unknown diagnosis despite routine genetic investigations sent for CMA between January 2009 and December 2012 |

**Figure S1: PRISMA** (**Preferred Reporting Items for Systematic Reviews and Meta-Analyses) Flow Diagram for Meta-analysis of Diagnostic Sensitivity and Rate of Clinical Utility of WGS, WES and CMA^S2^**

Records excluded (n = 1996): lacked measurement of diagnostic rate or clinical utility of WGS, WES or CMA.

Records identified through database searching (n = 2090)

Additional records identified through other sources (n = 3)

Studies included in quantitative synthesis (meta-analysis) (n = 21)

Studies included in qualitative synthesis (n = 37)

Full-text articles assessed for eligibility (n = 97)

Records screened (n = 2093)

Records after duplicates removed (n = 2093)

Full-text articles excluded (n = 60): Case reports, single disease studies, majority of probands >18 years of age, lacked measurement of diagnostic rate or clinical utility of WES, WGS or CMA, duplicative of an article included.

**Figure S2: Effects of disease type and proband age on the diagnostic (Dx) utility of genomic sequencing**. Moderate and severe rates of heterogeneity precluded quantitative analysis.


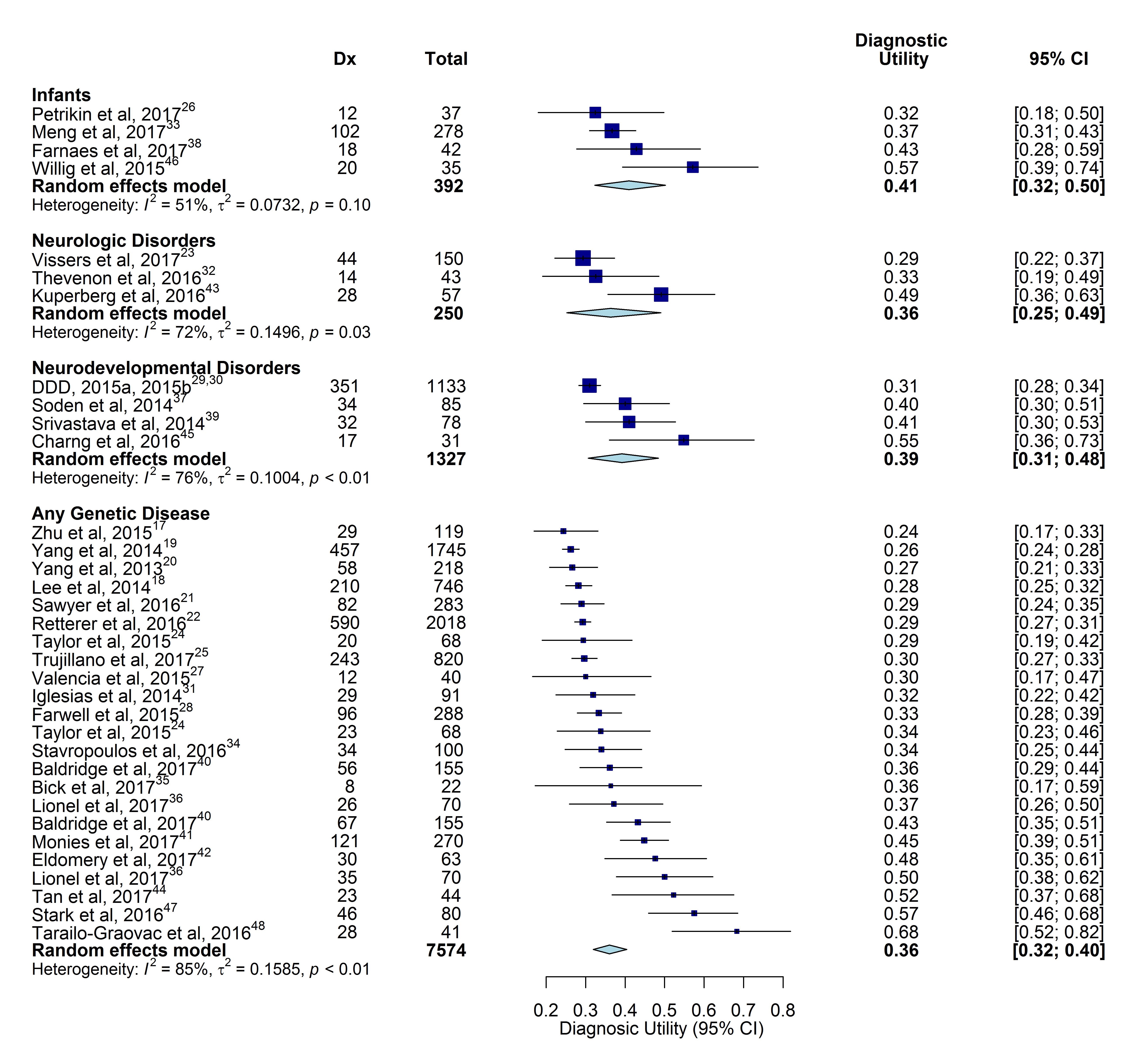


**Figure S3: Comparison of diagnostic (Dx) utility of singleton and trio genomic sequencing.** Severe heterogeneity within groups precluded quantitative analysis.


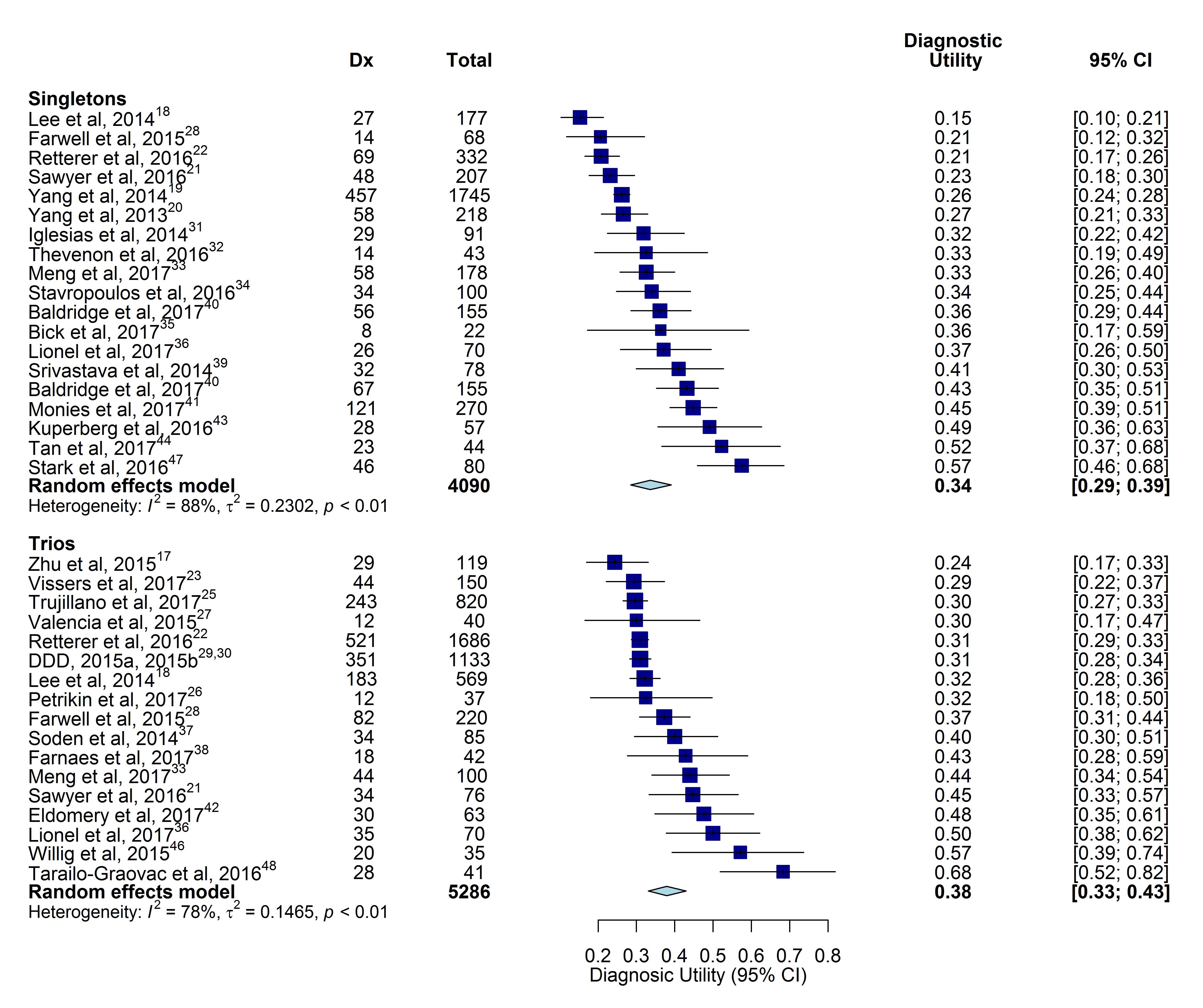

Supplement: Supplementary file 1 — Supplemental Material [file 41525_2018_53_MOESM1_ESM.docx]
